# Miscellaneous Notices

**Published:** 1854-07

**Authors:** 


### MISCELLANEOUS NOTICES.

*Prize of One Hundred Dollars Offered.*—It will be seen by the following communication that the "Mississippi Valley Association of Dental Surgeons," have offered a prize of one hundred dollars for the best Essay on Dental Surgery, to be adjudged by a committee appointed for the purpose. This is right, and we trust that an essay worthy of so handsome a prize will be presented. There is, however, in the resolution making the appropriation a want of definiteness with regard to the subject, which may render it difficult for the competitors to meet the views of the association. What is meant by "Dental Surgery?" Is it intended that the essay shall cover the whole field of this specialty, or only treat upon a few of its branches? or is it designed merely to set forth its claims to usefulness as compared with the other specialties of general medicine? The subject, it seems to us, should have been stated more definitely. Were we disposed to compete for the prize, we should be at a loss to know where to begin, or where to leave off. We trust, however, that the liberal offer of the association will elicit a paper that shall be creditable to itself, to the writer and to the profession.—*Eds.*

For the American Journal of Dental Science.

Messrs. Editors:—It has become my duty to request the insertion, in the Journal, of the following extract from the proceedings of the "Mississippi Valley Association of Dental Surgeons:"

"1st. *Resolved,* That this association will award one hundred dollars, to the author of the best essay, (not previously published,) on dental surgery, *adapted to popular circulation;* to contain not less than fifty, nor more than seventy-five pages duodecimo; the copy-right to be the property of the society. Said essay to be approved by a committee, and by it to be printed and issued to the members of the society, and receive the approval of the association before it be published for general circulation.

"2d. *Resolved,* That a committee of five be appointed, by ballot, to examine the essays presented, and report at the next meeting. Essays competing for said award to be forwarded to the chairman of the committee, as early as January 1st, 1855.

"Election of said committee was had, and the following gentlemen appointed: James Taylor, W. H. Goddard, A. Berry, A. M. Leslie and J. Taft.

"*Note*—Authors sending essays under the above resolutions, will accompany them with their full signature and address, which they will enclose in a sealed envelop, which will be opened should the accompanying essay prove the successful one; otherwise, it will be returned with the manuscript."

Geo. Watt,
*Cor. Sec., M. V. A. D. S.*

*Theory of the Circulation.*—Every one who reads the medical journals knows, that for some time, what is known as Mrs. Willard's Theory of the Circulation, has been stoutly defended by some medical men in the southwest. Dr. Cartwright, mounted on his mutilated alligator, is ready to charge on any one who controverts this notion. One of the most remarkable articles, however, to which this discussion has given rise, appears in the Nashville Journal of Medicine and Surgery for the present month.

We do not intend to comment on this paper at any length, but simply to expose the fallacy of a few of its fundamental propositions. The first of these is, that caloric is generated *in the lungs* by the combination of carbon and oxygen. "This," says the author, "is probably undisputed at the present time." So far from its being undisputed, this hypothesis has, by common consent, been set aside. We do not know of a single physiologist of any note, who continues to hold this opinion. It has been disproved in every possible manner—chemically, physically, vitally—and any theory built upon it must necessarily fall to the ground.

The author of the article to which we allude does not stop here, but goes on to show that the heat so generated will produce sufficient expansive force to carry on the circulation. *"All anatomists,"* he writes, *"agree that the lungs are in vacuo."* He then states the point at which water boils *in vacuo,* shows that it is below that of the healthy human body, and infers, therefore, that the fluid parts of the blood generate vapor enough to drive the vital fluid, with a sort of explosive force, out of the lungs.

It is astonishing how the ardent advocates of a theory over-leap every thing which lies between them and their conclusions. That is a novel sort of vacuum, indeed, which we find in the lungs. Outside, upon all the parietes of the chest, air is pressing, and inside, a column of air is constantly descending through the respiratory passages, mingling with that already in the lungs, and passing out to give place for a purer column, which is to enter at the next inspiration. Every little blood-vessel feels this pressure through the delicate mucous envelop which covers it. Every film of blood in the walls of the cells floats past this revivifying fluid, and interchanges gases with it as it goes, surrendering its carbonic acid and receiving oxygen.

The author has been deceived by the looseness of anatomical phrases. There is no air in the cavity of the *pleura,* and therefore the lungs fit closely to the thorax in all its movements. They are put into their movable box air-tight, but they are no more in a vacuum than the stuffing-box of a steam engine, which fits steam-tight around the piston-rod.

*The Dental News Letter.*—This journal complains, that in our April number we copied an article or two from it without giving due credit. Now accidents of this kind will occasionally happen, from hasty proof-reading and sometimes from neglect on the part of the printer to attend to the corrections made.

There is, however, no just ground of complaint in the present instance. The article by Dr. Evans, of Paris, is marked at the head of it, *continued,* and was duly credited in January, when the first part of it was copied. It was a mere oversight on the part of the printer that the name of the journal was not appended to the second part of the article in the April number. No reader, who had any common sense, would dream, for a moment, that the editors of this journal had any design to palm off the article in question as *original* matter, since it was put under the head of *selections* and credited, as we have already said, to its proper source in the previous number.

As to the second article, it appeared under the head of quarterly summary, which, of course, comprises only those facts and speculations which have *not* been published in our own journal. Owing to pressure of other business, the editors delegated the making up of the Quarterly Summary of Dental Progress to an inexperienced hand, who neglected to give credit to the *periodical* in which the article appeared, but fully acknowledged its *authorship* by giving Dr. White's name in full, and the summary of the first part of the article which appeared in the preceding number, was duly credited to the News Letter.

We have no disposition to trade upon a false bottom, and despise the meanness of appropriating the labors of another without due acknowledgment. We beg the editors of the News Letter to believe, that we would be just as much inclined to rob their money-drawer as to pilfer their articles. We were taught, from childhood, that the smallness of the larceny did not diminish the sin of the theft, and we have not forgotten the lesson.

*Ossification of the Pulp of a Tooth.*—We are indebted to Dr. F. H. Badger, of Nashville, Tenn., for a beautiful specimen of ossified dental pulp. In describing the circumstances connected with the case, the doctor says: "It was taken from the palatine root of the first right upper molar of my negro girl, aged about twenty, of full habit and healthy. She complained, for a few days, of severe pain in the tooth. Upon careful examination, I discovered, that though slightly decayed, the pulp was not exposed; the pain was but slightly increased on sounding it; no appearance of inflammation in the gums; the other teeth were sound. Refusing to have it extracted, she remained some days without relief. Finally, I was induced to drill into the pulp-cavity from the bottom of the decayed part, and apply arsenic. No pus escaped, but the patient was soon relieved. During the night the pain returned, with less violence, however, and at intervals. Some thirty-six hours after the application of arsenic, the cavity was sufficiently enlarged to extract the pulp, which, upon examination, was found to be interspersed with spicula of bone." The pulp was only removed from the two buccal roots which were filled, together with the cavity in the crown; the doctor finding it impossible to remove it from the palatine root. The patient not being relieved, the tooth was finally extracted, and after breaking off the crown and "enlarging to some extent, the canal," the pulp, in an ossified condition, was removed from the palatine root. This, the doctor enclosed in his letter to the senior editor, who intends placing it in the Museum of the Baltimore College of Dental Surgery.

*Brandy and Red Noses.*—Douglas Jerrold, in some remarks on English and Swedish brandy-drinking, gravely asks the question, whether the red noses of the English dram-drinkers are not due to the various adulterations introduced into the liquors by British dealers?

This reminds us of the verdict of a coroner's jury among the Mohawks, some years ago. An Indian had gone into Albany, one cold winter's day, and got very drunk. On his way home he became completely overcome, laid down and was frozen to death. His tribe was at that time much disposed to imitate the habits of white men, and accordingly held an inquest over the dead body. After a long pow-wow, they finally agreed upon the verdict, that the deceased had come to his death "by mixing too much *water* in his whiskey, which had frozen in him and killed him."

*The Late Dr. J. F. Flagg.*—We publish in the present number of the Journal, a biographical notice of the late Dr. J. F. Flagg, long an eminent member of the dental profession, written by a gentleman of Charleston, Mass. We had hoped to have accompanied the biography with a likeness of the subject of it, but have not been able to gratify our wishes in this respect.

Dr. G. G. Brewster, of Portsmouth, N. H., has also furnished us with an ably written biographical notice of Dr. F., paying a just tribute to his memory, and narrating, briefly, the incidents of his life, from his boyhood to the period of his decease.

*Dr. L. M. Cochran.*—The senior editor begs to acknowledge the receipt of a specimen of osseous union of two temporary teeth, and another of dental exostosis, from Dr. Cochran, dentist, of Matagorda, Texas. They shall, agreeably to his request, be placed in the Museum of the Baltimore Dental College. Both specimens are interesting, and well worthy of preservation. Contributions of this kind will always be thankfully received by the Faculty.

*Resignation.*—We are sorry to learn, that, in consequence of ill health, Professor Townsend, of the Philadelphia College of Dental Surgery, has felt it to be his duty to resign the Chair which he filled in that institution. This must be a serious loss to the school, but we are gratified to be able to say that the vacancy will be filled by a gentleman every way competent to discharge the duties of the Chair.

***Annual Commencement of the Baltimore College of Dental Surgery.***—The fourteenth annual commencement of this pioneer of dental colleges was held in the Assembly Room of the College Building, on the evening of the 1st of March. The spacious hall was filled at an early hour with a brilliant audience; an array of youth and beauty, which the graduates of the evening must long remember in association with these the first proud moments of professional dignity.

The exercises were opened with prayer by the Rev. Thomas H. Stockton; after which the following list of candidates for the degree of Doctor of Dental Surgery, with their places of residence and subjects of theses, was read by the Dean, Professor Austen.

Henry Fitch Bishop, orthodontia, Worcester, Mass.; William Chapman, qualification of dentists, Mt. Sidney, Va.; Lemuel Montgomery Cochran, mechanical dentistry, Matagorda, Texas; Thomas TsCharner DeGraffenreid, duties of the dentist, Clarksville, Va.; John William Derr, sympathy, Lititz, Penn.; Samuel Dearborn French, caries of the teeth, S. Chesterville, Me.; James Washington Grant, professional excellence, Botetourt Co., Va.; Edward Nathan Harris, temporary teeth, Portland, Me.; Willard Frederick Hawley, preservation of the teeth, Wirt Co., Va.; Montgomery Jeter, caries and its treatment, Roanoke Co., Va.; Edwin Turner Ligon, dental progress, Farmville, Va.; Henry Wainwright Mason, odontalgia, Boston, Mass.; Hugh Samuel Paisley, mercury and its preparations, Houston, Miss.; Albert Potter, mechanical dentistry, Blackstone, Mass.; Lloyd Quinby, pathogenia of a tooth and its treatment, Houston, Texas; William Thomas Russel, structure and development of the dental tissues, (prize thesis,) Canandaigua, N. Y.; Roger Forbis Taylor, aliment of man, Lockhaven, Penn.; Warner Archer Williams, effects of diseased teeth on the system, Richmond, Ala.

These gentlemen were then called up in order and received their diplomas at the hands of President Harris, who, after a brief reference to their exemplary diligence as students, urged upon them in a few pointed words their duty as practitioners of dental surgery. The frequent strains of music by the admirable band, and the more frequent tossing of beautiful bouquets, gave to this part of the exercises great animation and interest.

The valedictory was delivered by Dr. Wm. W. H. Thackston, of Farmville, Virginia. Graduating from these same halls "twelve years ago, when this was a new and untried enterprize," he now stood before them, proud to know that "its permanent existence and necessity had become fixed facts." He had no need to prove dentistry a "science," for its claims to that rank were now beyond dispute: therefore, passing that, he would present to the consideration of the graduating class, a few reflections upon the "obligations they had assumed as practitioners and as alumni of this institution." The brief limits of this notice will not permit us to dwell upon the eloquent manner in which the orator urged his hearers to engraft upon the *esse quam videri* motto of honest industry, the *suaviter in modo*

maxim of the courteous gentleman. The address has been printed, and we commend it to the profession.

The prize of $50 was then awarded by Professor Bond to WILLIAM THOMAS RUSSEL, of Canandaigua, N. Y., for his thesis upon the "structure and development of dental tissues;" after which the exercises of the evening were closed by the benediction.

*Trouble in the Wigwam.*—Two notorious homœopathic practitioners in this city have, it appears, been at loggerheads for some time in regard to the very important question of the possession of a diploma by one of them, and have at last appeared in the public prints through the medium of an advertisement.

The parties in the quarrel are Dr. McManus, one of the earliest practitioners of this species of quackery in our city, and one J. Schmidt, who may be known to some of our readers by a very silly onslaught he made a few years ago on our friend Dr. Bond. It appears that Dr. McManus had his doubts about the validity of the M. D. which Schmidt wrote after his name, as well he might on grounds far different from those upon which he based his objections. He accordingly erased Schmidt's name from the list of members of the American Institute of Homœopathy. The German, instead of regarding the act in its proper light, as a sort of compliment to his understanding, (for who would object to have his name struck out of the list of patients in a mad house?) took the matter in hand and had a committee appointed to look into it.

They ascertained that he had a document, which they call a diploma, from the Allentown Academy of Pennsylvania, an institution which has hitherto modestly concealed its existance from our benighted understandings, and appended the following resolution, which we copy as a choice morsel of homœopathic English. Its grammar has certainly reached the 30th dilution.

"*Resolved,* That Dr. J. Schmidt, of Baltimore, having a legal and valid diploma, from the Allentown Academy, of Pennsylvania, an institution which is now in existence, with the authority to confer medical (?) degrees, and we hereby acknowledge him to be entitled to the title of M. D."

Of the merits of the controversy we know nothing and care less, but so choice a curiosity in philology we could not consent to keep from our readers.

